# Supplementary material for: Mapping the evolution of fertility support policies in China: A content and instrumental analysis
Source: PLoS One. 2025 Oct 9;20(10):e0332137. doi: 10.1371/journal.pone.0332137 (PMC12510515; doi:10.1371/journal.pone.0332137)
Supplement: S1 Appendix — (ZIP) [file pone.0332137.s001.zip › S1 Appendix. 226 original policy documents/80-国务院关于印发国家基本公共服务体系“十二五”规划的通知(FBM-CLI-2-179549).docx]

国务院关于印发国家基本公共服务体系“十二五”规划的通知

发布部门： 国务院

发文字号：国发〔2012〕29号

发布日期：2012.07.11

实施日期：2012.07.11

时效性： 现行有效

效力级别： 国务院规范性文件

法规类别： 服务 国家计划

国务院关于印发国家基本公共服务体系“十二五”规划的通知

（国发〔2012〕29号）

各省、自治区、直辖市人民政府，国务院各部委、各直属机构：

现将《国家基本公共服务体系“十二五”规划》印发给你们，请认真贯彻执行。

国务院

2012年7月11日

国家基本公共服务体系“十二五”规划

目录

序言

第一章　规划背景

第一节　基本概念

第二节　规划范围

第三节　发展环境

第二章　指导思想和主要目标

第一节　指导思想

第二节　主要目标

第三章　基本公共教育

第一节　重点任务

第二节　基本标准

第三节　保障工程

第四章　劳动就业服务

第一节　重点任务

第二节　基本标准

第三节　保障工程

第五章　社会保险

第一节　重点任务

第二节　基本标准

第三节　保障工程

第六章　基本社会服务

第一节　重点任务

第二节　基本标准

第三节　保障工程

第七章　基本医疗卫生

第一节　重点任务

第二节　基本标准

第三节　保障工程

第八章　人口和计划生育

第一节　重点任务

第二节　基本标准

第三节　保障工程

第九章　基本住房保障

第一节　重点任务

第二节　基本标准

第三节　保障政策

第十章　公共文化体育

第一节　重点任务

第二节　基本标准

第三节　保障工程

第十一章　残疾人基本公共服务

第一节　重点任务

第二节　基本标准

第三节　保障工程

第十二章　促进城乡、区域基本公共服务均等化

第一节　促进城乡基本公共服务均等化

第二节　促进区域基本公共服务均等化

第十三章　增强公共财政保障能力

第一节　明确政府间事权和支出责任

第二节　完善转移支付制度

第三节　健全财力保障机制

第十四章　创新供给模式

第一节　建立多元供给机制

第二节　分类推进事业单位改革

第三节　鼓励社会力量参与

第十五章　规划实施

第一节　明确责任分工

第二节　加强监督问责

序言

“十二五”时期是我国全面建设小康社会的关键时期，是深化改革开放、加快转变经济发展方式的攻坚时期。建立健全基本公共服务体系，促进基本公共服务均等化，是深入贯彻落实科学发展观的重大举措，是构建社会主义和谐社会、维护社会公平正义的迫切需要，是全面建设服务型政府的内在要求，对于推进以保障和改善民生为重点的社会建设，对于切实保障人民群众最关心、最直接、最现实的利益，对于加快经济发展方式转变、扩大内需特别是消费需求，都具有十分重要的意义。

本规划根据《中华人民共和国国民经济和社会发展第十二个五年规划纲要》（以下简称“十二五”规划纲要）的有关要求编制，主要阐明国家基本公共服务的制度安排，明确基本范围、标准和工作重点，引导公共资源配置，是“十二五”乃至更长一段时期构建国家基本公共服务体系的综合性、基础性和指导性文件，是政府履行公共服务职责的重要依据。

　第一章　规划背景

　第一节　基本概念

基本公共服务，指建立在一定社会共识基础上，由政府主导提供的，与经济社会发展水平和阶段相适应，旨在保障全体公民生存和发展基本需求的公共服务。享有基本公共服务属于公民的权利，提供基本公共服务是政府的职责。

基本公共服务范围，一般包括保障基本民生需求的教育、就业、社会保障、医疗卫生、计划生育、住房保障、文化体育等领域的公共服务，广义上还包括与人民生活环境紧密关联的交通、通信、公用设施、环境保护等领域的公共服务，以及保障安全需要的公共安全、消费安全和国防安全等领域的公共服务。

基本公共服务标准，指在一定时期内为实现既定目标而对基本公共服务活动所制定的技术和管理等规范。

基本公共服务均等化，指全体公民都能公平可及地获得大致均等的基本公共服务，其核心是机会均等，而不是简单的平均化和无差异化。

基本公共服务体系，指由基本公共服务范围和标准、资源配置、管理运行、供给方式以及绩效评价等所构成的系统性、整体性的制度安排。

　第二节　规划范围

根据“十二五”规划纲要，为突出体现“学有所教、劳有所得、病有所医、老有所养、住有所居”的要求，本规划的范围确定为公共教育、劳动就业服务、社会保障、基本社会服务、医疗卫生、人口计生、住房保障、公共文化等领域的基本公共服务。

“十二五”规划纲要还明确了基础设施、环境保护两个领域的基本公共服务重点任务，包括：行政村通公路和客运班车，城市建成区公共交通全覆盖；行政村通电，无电地区人口全部用上电；邮政服务做到乡乡设所、村村通邮；县县具备污水、垃圾无害化处理能力和环境监测评估能力；保障城乡饮用水水源地安全等。这些内容分别纳入综合交通运输、能源、邮政、环境保护等相关“十二五”专项规划中，不在本规划中予以阐述。

本规划基本公共服务范围内涵（图略）

　第三节　发展环境

经过30多年的改革开放和发展建设，我国经济实力、综合国力和国际地位显著提高，人民生活明显改善。“十一五”以来，各地区、各有关部门认真贯彻落实党中央、国务院的决策部署，我国基本公共服务体系建设取得了显著成效。城乡免费义务教育全面实施，公共教育体系日趋完备，国民平均受教育年限达到9年。实施积极就业政策，初步建立起面向全体劳动者的公共就业服务体系。社会保险制度逐步由城镇向农村、由职工向居民扩展，保障水平逐步提高，城乡社会救助体系和社会福利体系基本形成。医药卫生体制改革深入推进，免费基本公共卫生服务项目全面实施，城乡基层医疗卫生服务体系逐步健全，国家基本药物制度初步建立。保障性安居工程加快建设，以廉租住房、公共租赁住房和农村危房改造等为主要内容的基本住房保障制度初步形成。基本实现县县有文化馆图书馆、乡乡有综合文化站，广播电视全面覆盖20户以上已通电自然村，公共博物馆、纪念馆、美术馆、公共图书馆、文化馆、科技馆等公共文化设施逐步向社会免费开放。全民健身稳步推进。公共服务财政投入显著增加。从总体上看，我国基本公共服务的制度框架已初步形成，人民群众上学、就业、就医、社会保障、文化生活等难点问题得到有效缓解。

但是，我国基本公共服务供给不足、发展不平衡的矛盾仍然十分突出，建立健全基本公共服务体系仍然面临许多困难和挑战。基本公共服务的规模和质量难以满足人民群众日益增长的需求；农村、贫困地区和针对社会弱势群体的基本公共服务尚未得到充分保障；体制机制有待于进一步完善，城乡区域间制度设计不衔接，管理条块分割，资源配置不合理，服务提供主体和提供方式比较单一，基层政府财力与事权不匹配，以及监督问责缺位等问题较为突出。必须深刻认识到，基本公共服务体系不健全，不仅难以保障发展成果惠及全民，不利于社会和谐稳定，而且还会制约经济社会健康协调可持续发展。

“十二五”时期，我国发展仍处于可以大有作为的重要战略机遇期，也是加快构建基本公共服务体系的关键时期。从需求看，工业化、信息化、城镇化、市场化、国际化深入发展，城乡居民收入水平不断提高，消费结构加快转型升级，各类公共服务需求日趋旺盛。从供给看，经济继续保持平稳较快发展，财政收入不断增加，基本公共服务财政保障能力进一步加强。从体制环境看，有利于科学发展的体制机制加快建立，教育、卫生、文化等社会事业改革深入推进，建立健全基本公共服务体系的体制条件不断完善。要牢牢抓住难得的历史机遇，顺应各族人民过上更好生活新期待，努力提升基本公共服务水平和均等化程度，推动经济社会协调发展，为全面建成小康社会夯实基础。

　第二章　指导思想和主要目标

　第一节　指导思想

高举中国特色社会主义伟大旗帜，以邓小平理论和“三个代表”重要思想为指导，深入贯彻落实科学发展观，把基本公共服务制度作为公共产品向全民提供，着力保障城乡居民生存发展基本需求，着力增强服务供给能力，着力创新体制机制，不断深化收入分配制度改革，加快建立健全符合国情、比较完整、覆盖城乡、可持续的基本公共服务体系，逐步推进基本公共服务均等化。

把基本公共服务制度作为公共产品向全民提供，是我国公共服务发展从理念到体制的创新。我国实行社会主义制度，公民都有获得基本公共服务的权利。保障人人享有基本公共服务是政府的职责，必须着眼制度设计、系统规划、整体推进，建立健全基本公共服务体系。基本要求是：

--以人为本，保障基本。从最广大人民群众的根本利益出发，立足我国社会主义初级阶段的基本国情，坚持尽力而为、量力而行，优先保障基本公共教育、劳动就业服务、社会保险、基本社会服务、基本医疗卫生、人口和计划生育、基本住房保障、公共文化体育等服务的提供，随着经济社会发展逐步扩大范围和提高标准。

--政府主导，坚持公益。牢牢把握基本公共服务的公益性质，明确政府的主体责任，完善公共财政体系，科学划分各级政府基本公共服务事权与支出责任，健全地方政府为主、统一与分级相结合的公共服务管理体制。加强立法、规划、投入、监管和政策支持，有效促进公平公正。

--统筹城乡，强化基层。打破行业分割和地区分割，加快城乡基本公共服务制度一体化建设，大力推进区域间制度统筹衔接，加大公共资源向农村、贫困地区和社会弱势群体倾斜力度，实现基本公共服务制度覆盖全民。把更多的财力、物力投向基层，把更多的人才、技术引向基层，切实加强基层公共服务机构设施和能力建设，促进资源共建共享，全面提高基本公共服务水平。

--改革创新，提高效率。完善财政保障、管理运行和监督问责机制，形成保障基本公共服务体系有效运行的长效机制。创新基本公共服务供给模式，引入竞争机制，积极采取购买服务等方式，形成多元参与、公平竞争的格局，不断提高基本公共服务的质量和效率。

　第二节　主要目标

今后一个时期，要把建立健全基本公共服务体系作为完善保障和改善民生制度安排、加快构建再分配调节机制的重大任务，并与全面建设小康社会战略目标和任务紧密衔接。“十二五”时期的主要目标是：

--供给有效扩大。政府投入大幅增加，基本公共服务预算支出占财政支出比重逐步提高。基本公共服务国家标准体系和标准动态调整机制逐步健全，各项制度实现全覆盖。创新公共服务供给方式，实现提供主体和提供方式多元化。

--发展较为均衡。资源布局更趋合理，优质资源共享机制加快建立，县（市、区）域内基本公共服务均衡发展基本实现，农村和老少边穷地区基本公共服务水平明显提高。

--服务方便可及。以基层为重点的基本公共服务网络全面建立，设施标准化和服务规范化、专业化、信息化水平明显提高，城乡居民能够就近获得基本公共服务。

--群众比较满意。城乡居民基本公共服务需求表达机制有效建立，服务成本个人负担比率合理下降，绩效评价和行政问责制度比较健全，社会满意度不断提高。

经过努力，“十二五”时期，覆盖城乡居民的基本公共服务体系逐步完善，推进基本公共服务均等化取得明显进展；到2020年实现全面建设小康社会奋斗目标时，基本公共服务体系比较健全，城乡区域间基本公共服务差距明显缩小，争取基本实现基本公共服务均等化。

　第三章　基本公共教育

国家建立基本公共教育制度，保障所有适龄儿童、少年享有平等受教育的权利，提高国民基本文化素质。

“十二五”时期，政府提供如下基本公共教育服务： ◆为适龄儿童、少年提供免费九年义务教育，为农村义务教育阶段寄宿生提供免费住宿，并为家庭经济困难寄宿生提供生活补助； ◆为贫困地区农村义务教育学生实施营养改善计划； ◆为农村学生、城镇家庭经济困难学生和涉农专业学生提供免费中等职业教育； ◆为家庭经济困难学生接受普通高中教育提供资助； ◆为家庭经济困难儿童、孤儿和残疾儿童接受学前教育提供资助。

　第一节　重点任务

重点巩固提高九年义务教育，基本普及高中阶段教育和学前一年教育，完善以政府为主导、多种方式并举的家庭经济困难学生资助政策，建立健全基本公共教育服务体系。

--九年义务教育。巩固九年义务教育普及成果，全面提高义务教育的质量和水平，着力推进义务教育均衡发展。统筹规划学校布局，推进义务教育学校标准化建设。保留必要的村小学和教学点，加强农村中小学寄宿制学校建设。提高农村义务教育阶段家庭经济困难寄宿生的生活费补助标准。将义务教育阶段的孤儿寄宿生全面纳入生活补助范围。公共教育资源重点向农村、边远、贫困、民族地区和革命老区倾斜，实行县（市、区）域内城乡中小学教师编制和工资待遇同一标准，以及教师、校长交流制度，逐步取消义务教育阶段重点校和重点班。以流入地全日制公办中小学为主，保证农民工随迁子女平等接受义务教育，并研究制定接受义务教育后在当地参加升学考试的办法。完善城乡义务教育学校的资源共建共享和对口交流支援制度。实施农村义务教育学生营养改善计划。巩固民族地区义务教育普及成果，推进双语教学。提高中小学教育信息化水平。全面实施素质教育，推进课程和教学方法改革，建立国家义务教育质量基本标准和监测制度，切实减轻中小学生课业负担。提高义务教育师资队伍能力水平，加强民族地区双语教师队伍建设。

--高中阶段教育。加强政府统筹，促进普通高中和中等职业教育协调发展。推动普通高中多样化发展，促进办学体制多元化，扩大优质资源。建立普通高中家庭经济困难学生国家资助制度。大力发展中等职业教育，坚持以服务为宗旨、以就业为导向，学校教育与职业培训并举，完善产学合作机制，全面推行工学结合、校企合作、顶岗实习的职业教育人才培养模式。加强职业教育教师队伍建设，鼓励技能型人才到职业学校从教。加强中等职业教育基础能力建设，建立健全职业教育质量保障体系。实行中等职业教育农村学生、城市家庭经济困难学生和涉农专业学生免学费政策，逐步实行中等职业教育免费制度。

--普惠性学前教育。建立政府主导、社会参与、公办民办并举的办园体制，构建覆盖城乡、布局合理的学前教育公共服务体系。为家庭经济困难儿童、孤儿和残疾儿童接受学前教育提供资助。大力发展公办幼儿园，鼓励优质公办幼儿园举办分园或合作办园。鼓励社会力量举办幼儿园，积极扶持民办幼儿园特别是面向大众、收费较低的普惠性民办幼儿园发展，采取政府购买、减免租金、以奖代补、派驻公办教师等方式，引导和支持民办幼儿园提供普惠性服务。根据居住区规划和居住人口规模，充分考虑农民工随迁子女接受学前教育的需求，配套建设城镇幼儿园。逐步完善县、乡、村学前教育网络，乡镇和大村独立建园，小村设分园或联合办园，人口分散地区举办流动幼儿园、季节班等。充分利用中小学布局调整富余的校舍和教师举办幼儿园（班）。积极发展民族地区学前双语教育。加强幼儿教师队伍建设。

　第二节　基本标准

加快建立健全基本公共教育服务国家标准体系。依据国家相关教育法律法规，为保障服务提供的规模和质量、明确工作任务的事权与支出责任、促进城乡区域均衡发展，制定“十二五”时期基本公共教育服务国家基本标准。

农村义务教育阶段中小学公用经费实行全国统一的基准定额。校舍建设、设备配置、师资配备、教学管理规范等具体标准，由教育部依法会同有关部门及国家标准化行政管理部门制定实施。

各省（区、市）应遵循实施国家基本标准，并可结合本地区实际情况，适当拓展基本公共教育服务范围和提高服务标准。

“十二五”时期基本公共教育服务国家基本标准

服务项目	服务对象	保障标准	支出责任	覆盖水平

九年义务教育

义务教育免费	适龄儿童、少年	免学费、杂费以及农村寄宿生住宿费，免费向农村学生提供教科书；农村中小学年生均公用经费标准，普通小学不低于500元，普通初中不低于700元	中央与地方财政按比例分担	目标人群覆盖率100%，九年义务教育巩固率达到93%

寄宿生生活补助	农村家庭经济困难寄宿学生	年生均补助小学1000元，初中1250元	地方政府负责，中央财政适当补助	目标人群覆盖率100%

农村义务教育学生营养改善	贫困地区农村义务教育学生	在寄宿生生活补助基础上，集中连片特殊困难地区每生每天营养膳食补助3元（每年在校时间按200天计）	地方政府负责，国家试点地区中央财政承担，其他地区中央财政适当补助	目标人群覆盖率100%

高中阶段教育

中等职业教育免费	农村学生、城镇家庭经济困难学生和涉农专业学生	免学费	中央与地方财政按比例分担	目标人群覆盖率100%，使高中阶段教育毛入学率达到87%

中等职业教育国家助学金	全日制在校农村学生及城市家庭经济困难学生	资助每生每年不低于1500元，资助两年	中央与地方财政按比例分担	目标人群覆盖率100%

普通高中国家助学金	家庭经济困难学生	平均资助每生每年1500元，地方结合实际在1000-3000元范围内确定	中央与地方财政按比例分担	目标人群覆盖率100%

学前教育

学前教育资助	家庭经济困难儿童、孤儿和残疾儿童	具体资助方式和标准由地方确定	地方政府负责，中央财政适当补助	目标人群覆盖率100%，学前一年毛入园率达到85%

　第三节　保障工程

根据建立健全基本公共教育体系的需要，实施一批保障工程，着力加强薄弱环节，改善薄弱学校办学条件，有效缩小城乡区域间教育发展差距。

--义务教育学校标准化建设工程。完善城乡义务教育经费保障机制，改造农村义务教育阶段薄弱学校，实现城乡中小学校舍、师资、设备、图书、体育场地基本达标。

--义务教育教师队伍建设工程。实施农村义务教育学校教师特设岗位计划和中小学教师国家级培训计划，加强农村学校薄弱学科教师队伍建设，建设农村边远艰苦地区教师周转宿舍。

--中等职业教育基础能力建设工程。扶持一批优质特色中等职业学校，改善实习实训设施条件，加强“双师型”教师队伍建设。

--民族教育发展工程。支持边境县和民族自治地方贫困县高中阶段学校建设，加强民族地区双语教师培训。

--农村学前教育推进工程。重点支持中西部贫困地区建设一批乡村幼儿园。

　第四章　劳动就业服务

国家建立劳动就业公共服务制度，为全体劳动者就业创造必要条件，加强劳动保护，改善劳动环境，保障合法权益，促进充分就业和构建和谐劳动关系。

“十二五”时期，政府提供如下劳动就业公共服务： ◆为全体劳动者免费提供就业信息、就业政策咨询、职业指导和职业介绍、就业失业登记等服务； ◆为就业困难人员和零就业家庭提供就业援助； ◆为失业人员、农民工、残疾人、新成长劳动力等提供职业技能培训和技能鉴定补贴； ◆为全体劳动者免费提供劳动关系协调、劳动人事争议调解仲裁和劳动保障监察执法维权等服务。

　第一节　重点任务

建立健全覆盖城乡的劳动就业公共服务体系，以高校毕业生、农村转移劳动力、城镇就业困难人员和零就业家庭为重点服务对象，全面提升就业全过程公共服务能力，努力创造平等就业机会，积极构建和谐劳动关系。

--就业服务和管理。完善并全面实施就业政策法规咨询、信息发布、职业指导和职业介绍、就业失业登记等免费服务，推进服务规范化和标准化，拓展服务功能。推进分类服务和管理，加快推行就业失业登记证实名制，尽快实现一人一证、全国通用。健全人力资源市场调查统计制度，建立全国就业信息监测制度，加强失业动态监测预警。完善就业援助政策，加大资金投入，完善税费减免、社会保险补贴、岗位补贴等办法，开发社区服务、养老服务、助残服务、交通协管、保洁、绿化等公益性岗位。加强公共就业服务网络建设，整合职业介绍和人才交流服务的公共资源，推动就业信息全国联网，提升就业创业和人才服务能力。

--职业技能培训。建立健全面向全体劳动者的职业培训制度，对城乡有就业要求和培训愿望的劳动者提供职业技能培训。对通过初次职业技能鉴定并取得职业资格证书或专项职业能力证书的，按规定给予一次性职业技能鉴定补贴。对未能升学的应届初高中毕业生等新成长劳动力普遍实行劳动预备制培训，给予培训费补贴，并对农村学员和城市家庭经济困难学员给予一定生活费补贴。加强职业技能培训经费统筹使用，提高效率和效益。加强职业技能培训能力建设，加大培训市场监管和资源整合力度，引导协调各类职业院校、培训机构有序开展职业技能培训，研究推进职业技能实训基地建设。

--劳动关系协调和劳动权益保护。全面推行劳动合同制度，着力提高小企业和农民工劳动合同签订率，扩大集体合同覆盖面。规范劳务派遣用工和企业裁员行为。全面推进实施劳动用工备案制度，加强对劳动用工的动态监管。健全企业薪酬调查和信息发布制度。完善企业工资决定机制和正常增长机制，积极稳妥推进工资集体协商工作。健全工资支付保障机制，完善最低工资和工资指导线制度，逐步提高最低工资标准。健全协调劳动关系三方机制，发挥政府、工会和企业作用。加强劳动保障监察执法力度，全面推进网格化、网络化管理，完善劳动案件办理协查制度。加强劳动人事争议调解仲裁服务体系建设，规范办案程序。建立健全重大集体劳动争议应急调处机制。

　第二节　基本标准

加快建立健全劳动就业公共服务国家标准体系。依据国家劳动就业服务相关法律法规，为保障劳动就业公共服务的规模和质量、明确工作任务的事权与支出责任，制定“十二五”时期劳动就业公共服务国家基本标准。

劳动就业公共服务机构设施建设、设备配置、人员配备、服务规范等具体标准，由人力资源社会保障部依法会同有关部门及国家标准化行政管理部门制定实施。

加强劳动标准体系建设。适时修订完善工作时间、休息休假、女职工和未成年工特殊劳动保护等标准，加强劳动定额标准管理。

各省（区、市）应遵循实施国家基本标准，并可结合本地区实际情况适当提高标准。

“十二五”时期劳动就业公共服务国家基本标准

服务项目	服务对象	保障标准	支出责任	覆盖水平

就业服务和管理	有就业需求的劳动年龄人口	免费享有就业政策法规咨询、职业供求信息、市场工资指导价位信息和职业培训信息、职业指导和职业介绍、就业和失业登记等服务	地方政府负责，中央财政适当补助	目标人群覆盖率达到100%

创业服务	有创业需求的劳动年龄人口	免费享有创业咨询指导、创业培训、创业项目推介，获得创业小额担保贷款贴息	地方政府负责，中央财政适当补助	为500万人次提供创业培训

就业援助	零就业家庭和符合条件的就业困难人员	免费享有公益性岗位配置和政策指导、就业困难人员和零就业家庭认定、就业岗位即时服务、就业培训等，城镇有就业需求的家庭至少有一人就业	地方政府负责，中央财政适当补助	帮助500万就业困难人员就业和再就业，动态消除零就业家庭

职业技能培训和技能鉴定	失业人员、农村转移就业劳动力、残疾人、新成长劳动力	失业人员、农村转移就业劳动力、残疾人等享有职业技能培训补贴，符合条件的新成长劳动力享有6-12个月的补贴性劳动预备制培训；符合条件的人员享有职业技能鉴定补贴	地方政府负责，中央财政适当补助	为1亿人次提供各类职业技能培训，培训后就业率不低于60%；为7500万人次提供技能鉴定

劳动关系协调	存在劳动人事关系的就业人员	免费享有劳动用工备案信息查询、劳动关系政策咨询、集体协商促进等服务	地方政府负责	企业劳动合同签订率达到90%，集体合同签订率达到80%

劳动保障监察	存在劳动人事关系的就业人员	免费享有法律咨询和执法维权服务	地方政府负责	监察案件结案率达到95%以上

劳动人事争议调解仲裁	存在劳动人事关系的就业人员	免费享有劳动人事争议调解和仲裁服务	地方政府负责	劳动人事争议仲裁结案率达到90%；50%以上案件在基层调解组织解决

　第三节　保障工程

按照整合、补缺、标准化的原则，因地制宜，统筹规划，加强就业、社会保险、劳动保障监察和调解仲裁等服务设施建设，形成覆盖城乡、功能齐全、布局合理、方便可及的就业服务网络，提高服务和管理的规范化、信息化、专业化水平。

--基层劳动就业和社会保障综合服务平台建设工程。全面加强县、乡两级服务设施（设备）建设，开展就业和职业技能培训、劳动关系协调、劳动保障监察和调解仲裁、人事人才、劳务输出等服务，提供社会保险参保登记、缴费、待遇核发、关系转移等经办服务。街道（乡镇）服务站、行政村（社区）服务窗口与其他公共服务设施共建共享。

--省、市（地）级人力资源市场建设工程。新建和改扩建一批省、市（地）级人力资源综合服务设施，改善综合就业和人力资源服务、劳动关系协调、劳动人事争议调解仲裁、劳动保障监察等服务的条件。

--就业失业动态监测和预警工程。建立健全覆盖全国的就业失业信息监测网络，完善就业信息统计和失业预警指标体系，开展就业需求预测，适时发布就业需求和失业预警信息。

　第五章　社会保险

国家建立基本养老保险、基本医疗保险、工伤保险、失业保险、生育保险等社会保险制度，保障公民在年老、疾病、工伤、失业、生育等情况下依法从国家和社会获得物质帮助的权利。

“十二五”时期，政府提供如下社会保险服务： ◆职工享有职工基本养老保险，农村居民享有新型农村社会养老保险，城镇居民享有城镇居民社会养老保险； ◆职工享有职工基本医疗保险，农村居民享有新型农村合作医疗，城镇居民享有城镇居民基本医疗保险； ◆职工享有失业保险、工伤保险、生育保险。

　第一节　重点任务

坚持广覆盖、保基本、多层次、可持续的方针，以增强公平性和适应流动性为重点，着力完善制度，扩大覆盖范围，逐步提高保障水平和统筹层次，建立健全覆盖城乡居民的社会保险体系。

--基本养老保险。以农民工、非公有制经济组织从业人员和灵活就业人员为重点，扩大职工基本养老保险覆盖面，将未参保集体企业退休人员全部纳入基本养老保险保障范围。推动机关事业单位养老保险制度改革。实现新型农村社会养老保险和城镇居民社会养老保险制度全覆盖，各地根据实际情况可以将两项制度合并实施。完善被征地农民基本生活保障制度，实行先保后征。实现基础养老金全国统筹，完善基本养老保险关系转移接续办法，逐步推进城乡养老保障制度有效衔接。建立健全与经济发展、工资增长和物价水平相适应的企业退休人员基本养老金正常调整机制，稳步提高新型农村社会养老保险和城镇居民社会养老保险基础养老金水平。

--基本医疗保险。扩大职工基本医疗保险制度覆盖范围，重点提高农民工、个体工商户和灵活就业人员参保率。巩固提高新型农村合作医疗参合率和城镇居民基本医疗保险参保率，逐步提高人均筹资标准和财政补助水平，鼓励有条件地区探索建立城乡统筹的居民基本医疗保险制度。全面推进基本医疗保险门诊统筹，将门诊常见病、多发病纳入保障范围，逐步提高门诊费用报销比例，基层医疗卫生机构门诊费用报销比例要明显高于医院。逐步提高医保基金最高支付限额和政策范围内住院费用报销比例，做好三项基本医疗保险待遇水平的衔接。提高儿童白血病、先天性心脏病等重大疾病医疗保障水平。探索建立重特大疾病保障机制，切实解决重特大疾病患者的因病致贫问题。完善基本医疗保险关系转移接续办法和医疗费用结算办法，全面实现统筹区域内和省内异地就医即时结算，逐步实现跨省异地就医结算。在确保基金安全和有效监管的前提下，鼓励以政府购买服务的方式，委托具有资质的商业保险机构经办各类医疗保障管理服务。

--工伤、失业和生育保险。健全预防、补偿、康复相结合的工伤保险制度，完善差别费率和浮动费率办法，适度提高待遇水平。将国有企业老工伤人员全部纳入工伤保险统筹管理。充分利用现有医疗和康复资源，加强工伤康复基地建设。完善失业保险制度，健全失业保险待遇正常调整机制，研究建立失业保险关系转移接续机制。完善生育保险制度，加强与基本医疗保险制度的衔接。以农民工、非公有制经济组织从业人员等为重点，扩大工伤、失业和生育保险覆盖面。积极探索建立农民意外伤害保障机制和覆盖城乡居民的生育保障机制。

　第二节　基本标准

加快建立健全社会保险服务国家标准体系。依据国家社会保险相关法律法规，为实现社会保险制度覆盖全民，并保障参保人员待遇水平，明确工作任务的事权与支出责任，制定“十二五”时期社会保险服务国家基本标准。

社会保险服务机构设施建设、设备配置、人员配备、服务规范等具体标准，由人力资源社会保障部、卫生部依法会同有关部门及国家标准化行政管理部门制定实施。

各省（区、市）应遵循实施国家基本标准，并可结合本地区实际情况适当提高标准。

“十二五”时期社会保险服务国家基本标准

服务项目	服务对象	保障标准	支出责任	覆盖水平

基本养老保险

职工基本养老保险	职工、无雇工的个体工商户、灵活就业人员	根据个人累计缴费年限、缴费工资、当地职工平均工资、个人账户金额、城镇人口平均预期寿命等因素确定基本养老金	用人单位缴纳一般不超过工资总额的20%，职工缴纳本人工资的8%，基金出现支付不足时由县级以上政府给予补贴	参保人数3亿人左右

新型农村社会养老保险	16周岁以上，未参加职工基本养老保险的农村居民	基础养老金不低于每人每月55元，并逐步提高标准	基础养老金由政府全额负担，个人缴费部分政府适当补贴	参保人数4.5亿人左右

城镇居民社会养老保险	年满16周岁（不含在校学生），不符合职工基本养老保险参保条件的城镇非从业居民	基础养老金不低于每人每月55元，并逐步提高标准	基础养老金由政府全额负担，个人缴费部分政府适当补贴	参保人数5000万人左右

基本医疗保险

职工基本医疗保险	职工、无雇工的个体工商户、灵活就业人员	政策范围内住院费用支付比例达到75%左右，最高支付限额达到当地职工年平均工资的8倍左右	用人单位缴纳工资总额的6%左右，职工缴纳本人工资的2%，基金出现支付不足时由县级以上政府给予补贴	参保人数2.6亿人左右

新型农村合作医疗	农村居民	政策范围内住院费用支付比例达到75%左右，最高支付限额达到当地农村居民年人均纯收入的8倍左右	个人和政府共同负担，各级财政的补助标准提高到年人均不低于360元，基金出现支付不足时由县级以上政府给予补贴	参合率稳定在90%以上

城镇居民基本医疗保险	城镇非从业居民	政策范围内住院费用支付比例达到75%左右，最高支付限额达到当地城镇居民人均可支配收入的8倍左右	个人和政府共同负担，各级财政的补助标准提高到年人均不低于360元，基金出现支付不足时由县级以上政府给予补贴	参保率稳定在90%以上

失业、工伤和生育保险

失业保险	职工	支付失业保险金、基本医疗保险费、丧葬补助金、抚恤金以及职业培训和职业介绍补贴等，失业保险金标准不低于城市居民最低生活保障标准	用人单位和职工按规定缴费，基金出现支付不足时由县级以上政府给予补贴	参保人数1.6亿人左右

工伤保险	职工	基金支付工伤医疗和康复、伤残、护理及工亡等待遇；用人单位支付停工留薪期的工资福利及护理待遇、5-6级伤残津贴待遇及一次性伤残就业补助金等	个人不缴费，用人单位根据行业差别费率和行业内费率档次缴费，基金出现支付不足时由县级以上政府给予补贴	参保人数2.1亿人左右

生育保险	职工	基金支付生育医疗费用和生育津贴，生育津贴按职工所在用人单位上年度职工月平均工资计发	用人单位缴费，基金出现支付不足时由县级以上政府给予补贴	参保人数1.5亿人左右

　第三节　保障工程

实施社会保险服务保障工程，改善服务设施条件，为城乡居民提供方便、快捷、高效的经办服务。

--省、市（地）级社会保障服务中心建设工程。新建和改扩建一批省、市（地）级社会保障服务设施，配置必要的设备，改善参保缴费、社会保险关系转移接续、待遇核发、社会保险档案管理、异地就医结算等经办服务条件。

--社会保障卡建设工程。逐步推行全国统一的社会保障卡，完成发放8亿张，覆盖60%以上人口，实现其在养老、医疗、工伤、失业、生育等社会保险的应用，并与就业服务、劳动关系、社会救助等信息共享。重点在国家、省、市（地）三级建设社会保障卡中心及其支持系统。

　第六章　基本社会服务

国家建立基本社会服务制度，为城乡居民尤其是困难群体的基本生活提供物质帮助，保障老年人、残疾人、孤儿等特殊群体有尊严地生活和平等参与社会发展。

“十二五”时期，政府提供如下基本社会服务： ◆为城乡困难群体提供最低生活保障和专项救助； ◆为农村五保对象提供吃、穿、住、医、葬方面的生活照顾和物质帮助； ◆为自然灾害受灾人员提供救助； ◆为城市生活无着的流浪乞讨人员提供救助； ◆为残疾人、孤儿、精神病人等特殊群体提供福利服务； ◆为老年人提供基本养老服务； ◆为优抚安置对象提供优待抚恤和安置服务； ◆为城乡居民免费提供婚姻登记服务； ◆为身故者提供基本殡葬服务。

　第一节　重点任务

着力健全以城乡最低生活保障制度为核心，以农村五保供养、自然灾害救助、医疗救助、流浪乞讨人员救助制度为主要内容，以临时救助制度为补充的社会救助体系。以扶老、助残、救孤、济困为重点，逐步拓展社会福利的保障范围，推动社会福利由补缺型向适度普惠型转变，逐步提高国民福利水平。加强优抚安置工作。

--社会救助。完善城乡最低生活保障制度，健全低保标准动态调整机制。采取多种措施提高老年人、残疾人、未成年人和重病患者的保障水平。建立低收入家庭认定体系，健全收入核查制度。加强城乡低保与最低工资、失业保险和扶贫开发等政策的衔接。将专项救助逐步延伸至低保边缘家庭，重点解决其医疗、教育、住房等方面的困难。加强医疗救助与基本医疗保险制度的衔接，逐步实行诊疗费用即时救助，降低医疗救助起付线，有条件的地方可以取消医疗救助起付线。健全自然灾害监测预警、评估调查、信息发布、应急救援和应急物资储备体系，完善救助技术标准和补助项目。完善临时救助制度。加强城市生活无着的流浪乞讨人员救助管理，加大流浪未成年人保护力度。

--社会福利。建立健全孤儿保障体系，合理确定孤儿养育标准，建立自然增长机制。拓展孤儿安置渠道，鼓励家庭养育。扩大福利机构收养能力。加强贫困和重度精神疾病患者收养和治疗服务。推动婚姻登记标准化和全国信息联网，推行婚姻免费登记。有条件的地方可向城乡基本生活困难家庭发放基本殡葬服务补贴，提供遗体运送、火化和绿色安葬等服务。加快实施免费地名公共服务。依托社区综合服务平台，为社区居民提供公益便民利民社区服务。

--基本养老服务。适应人口老龄化趋势，有条件的地方可发放高龄老年人生活补贴和家庭经济困难的老年人养老服务补贴。将符合条件的农村老人全部纳入农村五保供养范围，实行分散供养与集中供养相结合，适度提高供养标准。建立健全养老服务体系，鼓励居家养老，拓展社区养老服务功能，增强公益性养老服务机构服务能力，鼓励通过公建民营、民办公助等方式引导社会资本参与养老服务机构建设和管理运行。

--优抚安置。全面落实优抚对象各项优待政策，确保军人的抚恤优待与经济和社会发展相适应。实施残疾军人辅具改造。改善优抚设施条件，健全孤老优抚对象和重残退役军人集中供养制度。落实退役士兵安置改革各项政策，组织引导符合条件的退役士兵免费参加职业教育和技能培训。

　第二节　基本标准

加快建立健全基本社会服务国家标准体系。依据国家基本社会服务相关法律法规，为保障基本社会服务的规模和质量，明确工作任务的事权与支出责任，制定“十二五”时期基本社会服务国家基本标准。

各类基本社会服务机构资质认定、设施建设、设备配置、人员配备、服务规范以及服务对象资格认定等具体标准，由民政部依法会同有关部门及国家标准化行政管理部门制定实施。

各省（区、市）应遵循实施国家基本标准，并可结合本地区实际情况适当提高标准。

“十二五”时期基本社会服务国家基本标准

服务项目	服务对象	保障标准	支出责任	覆盖水平

社会救助

最低生活保障	家庭人均收入低于当地最低生活保障标准的城乡居民	保障标准按照能维持当地居民基本生活所必需的吃饭、穿衣、用水用电等费用确定，年均增长按国家“十二五”规划纲要确定的目标实施	地方政府负责，中央财政对困难地区适当补助	目标人群覆盖率100%

自然灾害救助	因自然灾害致使基本生活困难的人员	灾后12小时内基本生活得到初步救助	中央和地方政府共同负责	目标人群覆盖率100%

医疗救助	最低生活保障家庭、五保户以及低收入重病患者、重度残疾人、低收入家庭老年人等特殊困难群体	医疗救助起付线逐步降低或取消，政策范围内住院自负费用救助比例原则上不低于50%	地方政府负责，中央财政对困难地区适当补助	目标人群覆盖率100%

流浪乞讨人员生活救助	城市生活无着的流浪乞讨人员	免费享有临时基本食物、住处、急病救治、返乡及安置服务	县级以上政府负责	目标人群覆盖率100%，城区均设有标准的救助机构

流浪未成年人救助保护	流浪未成年人	免费享有生活照料、教育和职业培训、医疗救治、行为矫治、心理辅导、权益保护、返乡及安置等服务	县级以上政府负责	目标人群覆盖率100%，城区均设有标准的救助机构

社会福利

孤儿养育保障	失去父母、查找不到生父母的未成年人	孤儿基本生活最低养育标准由各地按不低于当地平均生活水平的原则合理确定，机构养育标准高于散居养育标准	地方政府负责，中央财政按照一定标准给予补助	目标人群覆盖率100%，新增孤儿养育床位20万张

农村五保供养	无劳动能力、无生活来源又无法定赡养、抚养、扶养义务人，或者法定赡养、抚养、扶养义务人无赡养、抚养、扶养能力的老年、残疾或者未满16周岁的村民	不低于当地村民的平均生活水平，并根据当地村民平均生活水平的提高适时调整，由地方政府确定	地方政府负责，中央财政对困难地区适当补助	目标人群覆盖率100%，集中供养能力达到50%以上

殡葬补贴	推行火葬地区不保留骨灰者和低收入家庭身故者的家庭	不保留骨灰者骨灰撒海等服务免费；有条件的地方为低收入家庭身故者遗体运送、火化以及安葬等提供补贴	地方政府负责	使火化率提高到50%

基本养老服务

基本养老服务补贴	家庭经济困难且生活难以自理的失能半失能65岁及以上城乡居民	有条件的地方根据老年人身体状况和家庭收入情况评估，确定补贴标准	地方政府负责	目标人群覆盖率50%以上

优抚安置

优待抚恤	享受国家抚恤补助的优抚人员	不低于当地平均生活水平	中央和地方政府分级负担	目标人群覆盖率100%

重点优抚对象集中供养	孤老和生活不能自理的抚恤优待对象	不低于当地平均生活水平	中央和地方政府共同负责	目标人群覆盖率100%

退役军人安置	退役军人	自主就业的，在领取退役金后，享受扶持就业优惠政策；其他分别采取安排工作、退休、供养等方式予以安置	中央和地方政府共同负责	目标人群覆盖率100%

　第三节　保障工程

按照应保尽保、应助尽助的要求，实施一批基本社会服务保障工程，提升基本社会服务水平。

--低收入家庭认定体系建设工程。结合建立收入信息监测系统，指导地方通过资源整合，加强低收入家庭收入核定工作机构及能力建设，逐步建立居民家庭经济状况核对信息系统。

--综合防灾减灾工程。重点推进国家自然灾害四级应急救助指挥系统、救灾物资储备库及综合应急避难场所等建设，加强社区减灾工作，开展防灾减灾专业人员特别是灾害信息员和志愿者队伍培训。

--孤残儿童保障服务工程。推进儿童福利机构建设，配备必要的专业救治和康复设施，培养培训2万名具有资质的孤残儿童护理员。拓展流浪未成年人保护设施功能，发挥庇护救助作用。

--养老服务体系建设工程。充分利用现有资源，加快专业化的老年养护机构和社区日间照料中心建设。增加养老床位300多万张，每千名老年人拥有养老床位数达到30张。支持有需求的失能老年人实行家庭无障碍设施改造。培养培训具有资质的专业养老服务人员。

　第七章　基本医疗卫生

国家建立基本医疗卫生制度，为城乡居民提供安全、有效、方便、价廉的基本医疗卫生服务，切实保障人民群众身体健康。

“十二五”时期，政府提供如下基本医疗卫生服务： ◆为城乡居民免费提供居民健康档案、健康教育、预防接种、传染病防治、儿童保健、孕产妇保健、老年人保健、高血压等慢性病管理、重性精神疾病管理、卫生监督协管等国家基本公共卫生服务； ◆实施国家免疫规划，艾滋病和结核病、血吸虫病等重大传染病防治，农村妇女住院分娩补助、适龄妇女宫颈癌乳腺癌检查等重大公共卫生项目； ◆实施国家基本药物制度，基本药物全部纳入基本医疗保障药物报销目录，并实行零差率销售； ◆为公众安全用药提供保障，确保药品质量和安全。

　第一节　重点任务

按照人人享有基本医疗卫生服务的目标要求，加快建立健全公共卫生服务体系、城乡医疗服务体系、药品供应和安全保障体系，提高基本医疗卫生服务的公平性、可及性和质量水平。

--公共卫生服务。全面实施国家基本公共卫生服务项目，逐步提高人均基本公共卫生服务经费标准。实施国民健康行动计划，根据经济社会发展水平和疾病防治工作需要，逐步增加重大公共卫生服务项目。完善重大疾病防控、计划生育、妇幼保健等专业公共卫生服务网络，提高对严重威胁人民健康的传染病、慢性病、地方病、职业病和出生缺陷等疾病的监测、预防和控制能力。完善卫生监督体系，建立食品安全标准及风险评估、监测预警、应急处置体系和饮用水卫生监督监测体系。依托县级医院实施农村院前急救网络建设。加强突发公共事件紧急医学救援能力和突发公共卫生事件监测预警、应急处理能力建设。积极发展中医预防保健服务。

--医疗服务。完善区域卫生规划。按照“大病不出县”、“小病不出社区”的要求，加强以县级医院为龙头、乡镇卫生院和村卫生室为基础的农村三级医疗卫生服务网络建设，健全以社区卫生服务为基础，社区卫生服务机构、医院和预防保健机构分工协作的城市医疗卫生服务体系。扩大城乡医院对口支援力度，推行乡村卫生服务一体化管理。加快建立分级诊疗、双向转诊和全科医生首诊制度。巩固和完善国家基本药物制度，推进基层医疗卫生机构综合改革，建立多渠道补偿机制，完善人事分配制度、考核和激励机制。积极推动公立医院改革，完善医院管理体制、法人治理机制、补偿机制和医疗机构分类管理制度。加强医疗服务监管，制定实施鼓励医疗卫生人才到基层服务的政策措施。推动形成多元化办医格局。统筹利用中西医卫生资源，加强中医（民族医）医疗服务机构能力建设，提高综合医院和专科医院中西医结合的服务能力。

--药品供应和安全保障。建立和完善以国家基本药物制度为基础的药品供应保障体系。政府办基层医疗卫生机构集中采购、统一配送、全部配备使用和零差率销售基本药物，逐步将村卫生室纳入基本药物制度实施范围，鼓励在非政府办基层医疗卫生机构实施基本药物制度，推动其他医疗机构优先使用基本药物。完善基本药物价格形成机制和调整机制，动态调整基本药物目录。鼓励提供与使用中医药。完善基本药物报销办法，逐步提高实际报销水平。全面提高国家药品标准，建立健全基本药物质量评价标准。完善药品检验检测体系，实行国家基本药物全品种覆盖抽验和全品种电子监管，提升对基本药物从生产到流通全过程追溯的能力。健全药品安全应急体系，强化快速通报和快速反应机制，完善药品不良反应监测和发布制度。

　第二节　基本标准

加快建立健全基本医疗卫生服务国家标准体系。依据国家医疗卫生领域相关法律法规，为保障基本医疗卫生服务的规模和质量，明确工作任务的事权与支出责任，促进城乡区域基本医疗卫生服务均衡发展，制定“十二五”时期基本医疗卫生服务国家基本标准。

医疗卫生机构设施建设、设备配置、人员配备、服务规范和药品生产流通等具体标准，由卫生部、食品药品监管局、中医药局依法会同有关部门及国家标准化行政管理部门制定实施。

各省（区、市）应遵循实施国家基本标准，并可结合本地区实际情况适当提高标准。

“十二五”时期基本医疗卫生服务国家基本标准

服务项目	服务对象	保障标准	支出责任	覆盖水平

基本公共卫生服务

居民健康档案	城乡居民	为辖区常住人口免费建立统一、规范的居民电子健康档案	地方政府负责，中央财政适当补助	规范化电子建档率达到75%以上

健康教育	城乡居民	免费享有健康教育宣传信息和健康教育咨询服务等	地方政府负责，中央财政适当补助	城乡居民具备健康素养的人数达到总人数10%

预防接种	0-6岁儿童和其他重点人群	免费接种国家免疫规划疫苗，在重点地区，对重点人群进行针对性接种	地方政府负责，中央财政适当补助	以街道（乡镇）为单位适龄儿童免疫规划疫苗接种率达到90%以上

传染病防治	法定传染病病人、疑似病人、密切接触者及相关人群	就诊的传染病病例和疑似病例及时得到发现登记、报告、处理，免费享有传染病防治知识宣传和咨询服务	地方政府负责，中央财政适当补助	传染病报告率和报告及时率达到100%；突发公共卫生事件相关信息报告率达到100%

儿童保健	0-6岁儿童	免费建立保健手册，享有新生儿访视、儿童保健系统管理、体格检查、生长发育监测及评价和健康指导	地方政府负责，中央财政适当补助	儿童系统管理率达85%以上

孕产妇保健	孕产妇	免费建立保健手册，享有孕期保健、产后访视及健康指导	地方政府负责，中央财政适当补助	孕产妇系统管理率达到85%以上

老年人保健	65岁及以上老年人	免费享有登记管理，健康危险因素调查、一般体格检查、中医体质辨识，疾病预防、自我保健及伤害预防、自救等健康指导	地方政府负责，中央财政适当补助	老年居民健康管理率达到60%

慢性病管理	高血压、糖尿病等慢性病高危人群	免费享有登记管理、健康指导、定期随访和体格检查	地方政府负责，中央财政适当补助	高血压和糖尿病患者规范化管理率达到40%以上

重性精神疾病管理	重性精神疾病患者	免费享有登记管理、随访和康复指导	地方政府负责，中央财政适当补助	重性精神疾病患者管理率达到70%

卫生监督协管	城乡居民	免费享有食品安全信息、学校卫生、职业卫生咨询、饮用水卫生安全巡查等服务与指导	地方政府负责，中央财政适当补助	目标人群覆盖率达到70%以上

药品供应和安全保障

基本药物制度	城乡居民	享有零差率销售的基本药物，并全部纳入基本医疗保障药物报销目录，逐步提高实际报销水平	地方政府负责，中央财政适当补助	覆盖所有政府办基层医疗卫生机构和村卫生室

药品安全保障	城乡居民	享有符合国家药品标准的药物	中央和地方政府共同负责	药品出厂检验合格率达到100%

　第三节　保障工程

实施一批基本医疗卫生服务保障工程，改善基础设施条件，健全服务网络，同步完善医疗卫生机构管理运行机制，为基本医疗卫生服务供给提供有力支撑。

--公共卫生服务体系建设工程。重点改善卫生监督、精神卫生、农村应急救治、食品安全等专业卫生服务机构基础设施条件，提高公共卫生服务和应急救治处置能力。

--医疗服务体系建设工程。推进基层医疗卫生机构标准化建设，提高县级医院（含中医医院）服务能力，加强省级妇儿专科医院、边远地区市（地）级综合医院、县级中医医院建设，使每个地级市都至少有一所综合医院达到三级医院水平，每个县（市、区）都至少有一所医院达到二级甲等医院水平。

--全科医生培养计划。加强以全科医生为重点的基层医疗卫生队伍建设，以三级综合医院和有条件的二级医院为临床培养基地，以社区卫生服务中心和专业公共卫生服务机构为实践基地，建设全科医生培养实训网络，通过转岗培训和规范化培训等多种途径培养15万名全科医生。

--医药卫生信息化建设工程。推进基层医疗卫生信息化建设。建设三级医院与县级医院远程医疗系统，加强公立医院信息化建设。

--药品安全保障基础设施建设工程。改善省、市（地）两级药品检验机构实验室条件，重点提升检验检测、认证检查和不良反应监测等药品安全技术支撑能力。

　第八章　人口和计划生育

国家建立人口和计划生育基本服务制度，为城乡居民提供计划生育、优生优育、生殖健康以及人口和计划生育信息等服务。

“十二五”时期，政府提供如下人口和计划生育基本服务： ◆为育龄人群免费提供避孕药具和避孕、节育技术服务； ◆为符合条件的育龄夫妇免费提供再生育技术服务； ◆为城乡居民免费提供计划生育、优生优育、生殖健康等科普宣传教育和咨询服务； ◆为符合条件的计划生育家庭提供奖励扶助。

　第一节　重点任务

坚持计划生育基本国策，以计划生育服务和计划生育利益导向为重点，完善人口和计划生育服务体系，保障城乡育龄人群身心健康，促进人口长期均衡发展。

--计划生育服务。创新人口和计划生育服务理念和模式。增强基层服务机构服务能力，依法拓展服务范围，加大流动服务、上门服务工作力度。加强流动人口计划生育服务管理，建立流动人口现居住地计划生育技术服务保障机制。进一步落实计划生育技术服务项目免费制度，完善避孕药具发放等的服务管理办法。推进出生缺陷一级预防工作，实行孕前优生健康检查，将免费孕前优生健康检查试点覆盖到全国31个省（区、市）。加强出生人口性别比偏高综合治理，广泛宣传男女平等观念，制定实施有利于女孩健康成长和妇女发展的社会经济政策，在扶贫济困、慈善救助、贴息贷款、就业安排、项目扶持中对计划生育女儿户予以倾斜。探索建立计划生育公益金制度。推进人口和计划生育信息化建设。加强人口和计划生育科普知识宣传。

--计划生育奖励扶助。继续实施和完善农村部分计划生育家庭奖励扶助制度、“少生快富”工程和计划生育特别扶助三项制度，扩大范围并建立动态调整机制。完善独生子女父母奖励制度，探索建立独生子女父母老年扶助制度和长效节育奖励制度。

　第二节　基本标准

加快建立健全人口和计划生育基本服务国家标准体系。依据国家人口和计划生育相关法律法规，为保障服务提供的规模和质量，明确工作任务的事权与支出责任，制定“十二五”时期人口和计划生育基本服务国家基本标准。

计划生育服务机构设施建设、设备配置、人员配备、服务规范等具体标准，由人口计生委依法会同有关部门及国家标准化行政管理部门制定实施。

各省（区、市）应遵循实施国家基本标准，并可结合本地区实际情况适当提高标准。

“十二五”时期人口和计划生育基本服务国家基本标准

服务项目	服务对象	保障标准	支出责任	覆盖水平

计划生育服务

技术指导咨询	育龄人群	免费获取避孕药具，免费享有查环查孕经常性服务、术后随访服务及计划生育、优生优育、生殖健康科普、教育、咨询服务	免费避孕药具支出由中央财政全额负担，其他服务由地方政府负责，中央财政适当补助	本地常住人口目标人群覆盖率100%，流动人口目标人群覆盖率达到85%

临床医疗服务	育龄夫妇	免费享有避孕和节育的医学检查、计划生育手术、计划生育手术并发症和计划生育药具不良反应的诊断、治疗	地方政府负责，中央财政适当补助	避孕节育免费服务目标人群覆盖率100%

再生育技术服务	符合条件的育龄夫妇	免费享有再生育相关的医学检查、输卵（精）管复通手术	地方政府负责，中央财政适当补助	目标人群覆盖率100%

宣传服务	城乡居民	免费获取计划生育、优生优育、生殖健康等宣传品	地方政府负责，中央财政适当补助	家庭覆盖率达到90%

计划生育奖励扶助

独生子女父母奖励	实行计划生育、子女未满18周岁的夫妇	奖励费每对夫妇每年不低于120元	中央、地方、企事业单位共同负担	目标人群覆盖率80%以上

农村部分计划生育家庭奖励扶助	年满60周岁、只生育一个子女或两个女孩的农村计划生育家庭夫妇	奖励扶助金夫妇每人年均不低于960元	中央和地方财政按比例共同负担	目标人群覆盖率95%以上

“少生快富”	特定农牧区可生三个孩子而自愿少生一个或两个孩子，并已落实安全适宜长效节育措施的夫妇	一次性奖励每对夫妇不少于3000元	中央财政负担80%，地方财政负担20%	覆盖内蒙古、海南、四川、云南、甘肃、青海、宁夏、新疆和新疆生产建设兵团所有目标人群

计划生育家庭特别扶助	符合条件的死亡或伤残独生子女父母及节育手术并发症三级以上人员	根据不同情况，给予每人每月不低于135元、110元的扶助金；给予节育手术并发症一级、二级、三级人员适当补助	中央和地方财政按比例共同负担	目标人群覆盖率90%以上

　第三节　保障工程

--实施人口和计划生育服务体系建设工程。改造部分市（地）级、县级和乡（镇）中心站基础设施，更新、增配必要的计划生育流动服务车和相关设备，提高信息化水平，使每个县和中心乡镇都有一个符合国家标准的人口和计划生育服务机构。开展人口和计划生育队伍职业化、专业化建设。

　第九章　基本住房保障

国家建立基本住房保障制度，维护公民居住权利，逐步满足城乡居民基本住房需求，实现住有所居。

“十二五”时期，政府提供如下基本住房保障服务： ◆为城镇低收入住房困难家庭提供廉租住房或租赁补贴； ◆为城镇中等偏下收入住房困难家庭、新就业无房职工和城镇稳定就业的外来务工人员提供公共租赁住房； ◆为符合条件的棚户区居民实施住房改造； ◆为农村困难家庭危房改造提供补助。

　第一节　重点任务

加大保障性安居工程建设力度，增加保障性住房供应，加快解决城镇居民基本住房问题和农村困难群众住房安全问题，建立健全基本住房保障制度。

--廉租住房和公共租赁住房。保障性住房实行分散配建和集中建设相结合。集中建设保障性住房，要优先安排在交通便利、基础设施齐全、公共事业完备、就业方便的区域。健全廉租住房保障方式，实行实物配租和租赁补贴相结合。多渠道筹集廉租住房房源。完善租赁补贴制度，通过发放租赁补贴增强低收入家庭在市场上承租住房的能力。重点发展公共租赁住房，逐步使其成为保障性住房的主体，并逐步实现与廉租住房统筹建设、并轨运行。面向有一定支付能力的城镇中低收入住房困难家庭，适当发展经济适用住房和限价商品住房。

--棚户区改造。全面推进城市和国有工矿棚户区、中央下放地方煤矿棚户区、国有林区棚户区和国有林场危旧房、国有垦区危房改造。稳步推进非成片棚户区、零星危旧房改造。逐步开展基础设施简陋、建筑密度大、集中连片的城镇旧住宅区综合整治，稳步实施“城中村”改造，改善基础设施条件，完善居住功能。

--农村危房改造。继续推进农村危房改造，合理确定补助对象和标准，优先帮助住房最危险、经济最贫困农户解决住房安全问题。落实建设基本要求，强化工程质量安全管理，完善档案管理和产权登记，推动农村基本住房安全保障制度建设。推进游牧民定居工程建设，提高建设质量和规范化水平。

--保障性住房管理。加快基本住房保障立法工作，做好廉租住房、公共租赁住房和经济适用住房等各类保障性住房的政策衔接。鼓励各地依法建立保障性住房投资机构。研究建立全国性和区域性个人住房贷款担保体系，支持中低收入家庭改善住房条件。建立健全多部门联动的收入（财产）和住房情况动态监管机制，制定公平合理、公开透明的保障性住房配租政策和监管程序，严格规范准入、退出管理和租费标准。加强棚户区改造项目管理，推进市政基础设施和公共服务设施配套建设。实施能力建设工程，建立健全保障性住房管理服务机构，提升住房保障管理人员素质，加强规范化管理。建立全国住房保障基础信息管理平台，促进全国住房保障业务系统互联互通。

　第二节　基本标准

加快建立健全基本住房保障服务国家标准体系。依据基本住房保障有关政策规定，为保证保障性住房的供给规模和质量，明确工作任务的事权与支出责任，制定“十二五”时期基本住房保障服务国家基本标准。

基本住房保障对象的家庭收入（财产）标准、住房困难标准、租金标准和保障面积标准，由市（地）、县级政府在国家标准框架内结合当地实际确定，并实行年度动态管理。

基本住房保障管理服务机构的设施建设、设备配置、人员配备、服务规范等具体标准，由住房城乡建设部依法会同有关部门及国家标准化行政管理部门制定实施。

“十二五”时期基本住房保障服务国家基本标准

服务项目	服务对象	保障标准	支出责任	覆盖水平

廉租住房	城镇低收入住房困难家庭	享有实物配租的，人均住房建筑面积13m2左右，套型建筑面积50m2以内，租金标准由市、县政府确定；享有租赁补贴的，租赁补贴标准由市、县政府根据当地经济发展水平、市场平均租金、家庭经济承受能力等因素确定	市、县政府负责，省级政府给予资金支持，中央给予资金补助	增加廉租住房不低于400万套，新增发放租赁补贴不低于150万户

公共租赁住房	城镇中等偏下收入住房困难家庭、新就业无房职工、城镇稳定就业的外来务工人员	单套建筑面积以40m2左右的小户型为主，租金水平由市、县政府根据市场租金水平和供应对象的支付能力等因素确定	市、县政府负责，引导社会资金投入，省级政府给予资金支持，中央给予资金补助	增加公共租赁住房不低于1000万套

棚户区改造	符合条件的棚户区居民	实物安置和货币补偿相结合，具体标准由市、县政府确定（有国家标准的，执行国家标准）	政府给予适当补助，企业安排一定的资金，住户承担一部分住房改善费用	改造棚户区居民住房不低于1000万户

农村危房改造	居住在危房中的农村分散供养五保户、低保户、贫困残疾人家庭和其他贫困户	每户建筑面积一般控制在40-60m2，户均中央补助不低于6000元，地方补助标准自行确定	省级政府负总责，中央财政安排补助资金、省级财政给予资金支持、个人自筹等相结合	改造农村危房800万户以上

游牧民定居	未定居的游牧民	每户建筑面积不低于60m2（考虑家庭平均人口差异，内蒙古自治区户均50m2），户均中央补助3万元，户均地方配套1.6万元	省级政府负总责，中央财政安排补助资金、地方财政给予资金支持、个人自筹相结合	基本完成24.6万户游牧民的定居任务

　第三节　保障政策

进一步完善土地、财税、金融等政策体系，建立稳定投入机制，加大财政资金、住房公积金贷款、银行贷款的支持力度，引导社会力量参与保障性安居工程建设和运营。

--土地政策。在土地利用年度计划中要根据保障性安居工程建设需要，单独列出，做到应保尽保。依法收回的闲置土地、具备净地出让条件的储备土地和农用地转用计划指标，应优先保证保障性住房用地。

--财税政策。加大财政投入力度，完善财政投入方式。土地出让收益用于保障性住房建设和棚户区改造的比例不低于10%。地方政府债券优先用于保障性安居工程建设。住房公积金增值收益在提取贷款风险准备金和管理费用后，全部用于廉租住房和公共租赁住房建设。对保障性安居工程建设和运营给予税费优惠。其中，对廉租住房、公共租赁住房、经济适用住房及棚户区安置住房，免收各种行政事业性收费和政府性基金。

--金融政策。支持保险资金、信托资金、房地产信托投资基金等投资保障性安居工程建设和运营。支持符合条件的地方政府融资平台公司和其他企业发行企业（公司）债券、上市公司债券，多渠道筹集建设资金。鼓励商业银行按照风险可控的原则，发放公共租赁住房等保障性住房中长期贷款。支持符合条件的省级政府以及计划单列市、省会城市、地级市政府融资平台公司进行廉租住房、公共租赁住房和棚户区改造融资。

--价格政策。依据经济社会发展水平、保障对象的承受能力以及建设成本等因素，合理制定、调整保障性住房价格或租金标准。

　第十章　公共文化体育

国家建立公共文化体育服务制度，保障人民群众看电视、听广播、读书看报、进行公共文化鉴赏、参加大众文化活动和体育健身等权益。

“十二五”时期，政府提供如下公共文化体育服务： ◆向全民免费开放基层公共文化体育设施，逐步扩大公共图书馆、文化馆（站）、博物馆、美术馆、纪念馆、科技馆、工人文化宫、青少年宫等免费开放范围； ◆为全民免费提供基本的广播电视服务和突发事件应急广播服务； ◆为农村居民免费提供文化信息资源共享、电影放映、送书送报送戏等公益性文化服务； ◆加强文化遗产保护和综合利用； ◆为城乡居民参加全民健身活动提供免费指导服务。

　第一节　重点任务

围绕建设社会主义核心价值体系和满足城乡居民精神文化需求的要求，坚持公益性、基本性、均等性、便利性，建立健全公共文化服务体系，扩大公共文化产品和服务的供给。推进全民健身公共服务体系建设。

--公益性文化。继续实施文化惠民工程，以农村基层和中西部地区为重点，加快公共文化基础设施建设。推进建立公共电子阅览室和未成年人公益性上网场所。促进城乡基层公共文化服务资源的共建共享。逐步实现公共文化场馆向全社会免费开放。推动文化科技卫生“三下乡”、“送欢乐下基层”等活动制度化，充分发挥流动文化服务车、流动电影放映车作用。广泛开展社区文化、村镇文化、校园文化、家庭文化等群众性文化活动，积极开展面向农民工和残疾人等群体的公益性文化服务。完善公益性演出补贴制度。加大对地方特色和民族特色文化的支持力度。加大文化和自然遗产、非物质文化遗产保护力度，逐步提高面向公众开放、展示的水平。

--广播影视。加强农村基层广播电视和无线发射台站建设，全面解决20户以下已通电自然村“盲村”广播电视覆盖。加强直播卫星平台建设，在有线网络未通达、无线网络不能覆盖的农村地区开展直播卫星公共服务。提高少数民族语言广播影视节目译制、制作、播出及传播覆盖能力。继续推进农村电影数字放映，将观看爱国主义教育影片纳入中小学教育教学计划。鼓励电影企业深入城乡社区、厂矿等开展公益放映活动。积极推进国家应急广播体系建设。加强地面数字电视建设，逐步完成地面模拟信号向数字信号的转换，不断提高无线广播电视公共服务的质量和水平。

--新闻出版。广泛开展全民阅读活动，逐步扩大基本免费或低收费阅读服务范围。继续加强农家书屋和城乡阅报栏（屏）建设，合理规划布局建设农村和中小城市出版发行网点。推进公益性数字出版产品免费下载、阅读和使用。大力扶持少数民族出版物的翻译和出版，积极开展少数民族文字书报刊赠送活动。

--群众体育。加强基层公共体育设施建设。大力推动公共体育设施向社会开放，健全学校等企事业单位体育设施向公众开放的管理制度。全面实施全民健身计划，健全基层全民健身组织服务体系，扶持社区体育俱乐部、青少年体育俱乐部和体育健身站（点）等建设，发展壮大社会体育指导员队伍，大力开展全民健身志愿服务活动。积极推广广播体操、工间操以及其他科学有效的全民健身方法，广泛开展形式多样、面向大众的群众性体育活动。建立国家、省、市三级体质测定与运动健身指导站，普及科学健身知识，指导群众科学健身。推动落实国家体育锻炼标准，加强学生体质监测，制定残疾人体质测定标准，定期开展国民体质监测。

　第二节　基本标准

加快建立健全公共文化体育服务国家标准体系。依据国家文化体育相关法律法规，为保障服务的供给规模和质量，明确工作任务的事权与支出责任，促进城乡均衡发展，制定“十二五”时期公共文化体育服务国家基本标准。

各类公共文化体育设施布局、场馆建设、设备配置、人员配备、服务规范等具体标准，由文化部、广电总局、新闻出版总署、文物局和体育总局依法会同有关部门及国家标准化行政管理部门制定实施。

各省（区、市）应遵循实施国家基本标准，并可结合本地区实际情况适当提高标准。

“十二五”时期公共文化体育服务国家基本标准

服务项目	服务对象	保障标准	支出责任	覆盖水平

公益性文化服务

公共文化场馆开放	城乡居民	公共空间设施和基本服务项目免费，全年开放时间不少于10个月	中央和地方财政按比例共同负担	除文物建筑及遗址类博物馆外，各级文化文物部门归口管理的公共文化场馆全面向社会开放

公益性流动文化服务	城乡居民	免费享有影视放映、文艺演出、图片展览、图书销售和借阅、科技宣传为一体的流动文化服务；每个乡镇每年送4场地方戏曲；每学期中小学生观看两部爱国主义教育影片	地方政府负责，中央财政适当补助	基本建立灵活机动、方便群众的公益性流动文化服务网络，保障公益性演出场次

广播影视

农村广播电视	农村居民为主	无偿提供中央第一套广播节目、中央第一套和第七套电视节目及本省第一套广播电视节目等4套以上广播和电视节目服务，逐步增加节目套数和提高播放质量	中央和地方政府共同负责	基本实现所有通电行政村和自然村村村和户户通广播电视

农村电影放映	农村居民	行政村一村一月放映一场电影，每场财政补贴200元	中央和地方财政按比例共同负担	每年放映780万场公益电影

少数民族语言广播影视	主要少数民族地区居民	通过有线、无线或卫星等方式能够收听收看到本民族语言广播影视节目	中央和地方政府共同负责	覆盖藏、维、蒙、哈、朝、壮、傣等主要少数民族地区

应急广播	城乡居民	在突发公共事件发生前后及时获得政令、信息等服务	中央和地方政府共同负责	在全国范围内基本实现分层次、分类型、全方位立体覆盖

新闻出版

公共阅读服务	城乡居民	农村行政村建立农家书屋，图书不少于1500册，报刊20-30种，电子音像制品不少于100种（张），并及时更新；城市和乡镇主要街道、大专院校、居民小区等人流密集地点设公共阅报栏（屏），及时提供各类新闻和服务信息	中央和地方财政按比例共同负担	基本实现行政村村村有农家书屋，新增城乡公共阅报栏（屏）10万个，国民综合阅读率达到80%

民文出版译制	有文字的少数民族	可以获得本民族语言文字出版的、价格适宜的常用书刊、电子音像制品，政府给予出版物资助	中央和地方政府共同负责	每年选择不少于800种优秀国内外书刊、电子音像制品翻译成少数民族语言文字

盲文出版	盲人	可以获得价格适宜的盲文出版物，政府给予出版物资助	中央和地方政府共同负责	年生产盲文书刊1600种、70万册

文化遗产展示

文化遗产展示门票减免	未成年人、老年人、现役军人、残疾人和低收入人群	减免参观文物建筑及遗址类博物馆的门票	中央和地方财政分别负担	目标人群覆盖率100%

群众体育

体育场馆开放	城乡居民	有条件的公办体育设施（含学校体育设施）向公众开放，免费项目或有关收费标准由地方政府制定；开放时间与当地公众的工作时间、学习时间适当错开，不少于省（区、市）规定的最低时限，全民健身日免费开放，国家法定节假日和学校寒暑假期间，应当适当延长开放时间	地方政府负责，中央财政适当补助	可供使用的公共体育场地（含学校体育场地）占全国体育场地总数的比率达到53%左右

全民健身服务	城乡居民	免费享有健身技能指导、参加健身活动、获取科学健身知识等服务；免费提供公园、绿地等公共场所全民健身器材	地方政府负责，中央财政适当补助	经常参加体育锻炼人数比率达到32%以上

　第三节　保障工程

实施公共文化体育服务保障工程，健全服务网络，着力改善基层文化体育设施条件，有效提升公共文化体育服务能力。

--公共文化服务体系建设工程。继续推进广播电视村村通、文化信息资源共享、国家数字图书馆推广工程、公共电子阅览室建设计划、农村数字电影放映、农家书屋、西藏新疆等边疆民族地区广播电视覆盖工程和边疆地区少数民族新闻出版工作，实施地面数字电视覆盖和直播卫星广播电视公共服务建设，新建、改扩建一批市（地）级公共图书馆、文化馆、博物馆。

--传播体系建设工程。重点加强媒体传播能力、民族文字出版和民族语言广播、文化传播渠道、国家应急广播体系建设。

--文化和自然遗产保护工程。重点支持国家重大文化和自然遗产地、全国重点文物保护单位、大遗址、中国历史文化名城名镇名村保护设施建设，推进非物质文化遗产保护利用设施建设试点。做好历史档案和文化典籍保护整理工作。

--体育基本公共服务建设工程。重点支持县级公共体育场建设，加快建设一批面向群众、贴近基层的中小型全民健身中心和灯光球场，充分利用城市绿地、广场、公园等公共场所和适宜的自然区域建设全民健身活动设施。继续实施农民体育健身工程，改善农村公共体育设施条件。

　第十一章　残疾人基本公共服务

国家为残疾人提供适合其特殊需求的基本公共服务，营造残疾人平等参与的社会环境，为残疾人生活和发展提供稳定的制度性保障。

“十二五”时期，政府提供如下残疾人基本公共服务： ◆为符合条件的贫困残疾人参加社会保险按规定给予补贴； ◆为0-6岁残疾儿童免费提供抢救性康复； ◆为适龄残疾儿童、少年免费提供义务教育，并针对残疾学生的特殊需要适当提高补助水平； ◆为残疾人免费提供就业服务和就业援助； ◆为残疾人提供盲人阅读、聋人手语及影视字幕、特殊艺术、自强健身等公共文化体育服务； ◆为残疾人提供无障碍环境。

　第一节　重点任务

按照平等、参与、共享的原则，以重度残疾人、农村残疾人和残疾儿童为重点，优先发展社会急需、受益面广、效益好的残疾人基本公共服务，增强供给能力，健全残疾人社会保障体系和服务体系。

--残疾人社会保障。落实和完善贫困残疾人参加社会保险保费补贴政策，提高残疾人社会保险参保率和待遇水平。逐步将符合规定的残疾人医疗康复项目纳入基本医疗保险支付范围，逐步增加工伤保险职业康复项目。着力解决好重度残疾、一户多残、老残一体等特殊困难家庭的基本生活保障问题，做好低收入残疾人家庭生活救助。有条件的地方实施贫困残疾人生活补助和重度残疾人护理补贴制度。构建辅助器具适配体系，有条件的地方对重度残疾人适配基本型辅助器具给予补贴。

--残疾人基本服务。建立健全以专业康复和托养服务机构为骨干、社区为基础、家庭为依托的社会化残疾人康复、托养服务体系。加强残疾人服务设施建设，继续实施“阳光家园”计划，实施国家重点康复工程，建立残疾儿童抢救性康复救助制度。完善残疾学生助学政策，保障残疾学生和残疾人家庭子女免费接受义务教育，逐步实行残疾学生高中阶段免费教育，推进特殊教育学校标准化建设。加大残疾人就业促进和保护力度，开展多层次残疾人职业技能培训，为农村残疾人提供实用技术培训，落实残疾人按比例就业、安置残疾人单位税收优惠、残疾人个体就业扶持等政策。公共就业服务机构和残疾人就业服务机构免费为残疾人提供有针对性的职业介绍、职业指导等就业服务。将住房困难的城乡低收入残疾人家庭优先纳入基本住房保障制度。加强针对盲人和聋人特殊需求的公共文化服务，实行公共文化体育设施对残疾人优惠开放，扩大盲人读物出版规模。加快无障碍建设和改造，推进公共设施设备和信息交流无障碍，有条件的地方为有需求的贫困残疾人家庭无障碍改造提供补助。建立健全残疾预防体系。

　第二节　基本标准

加快建立健全残疾人基本公共服务国家标准体系。依据国家残疾人事业相关法律法规，为保障残疾人基本公共服务的规模和质量，明确工作任务的事权与支出责任，缩小残疾人生活状况与社会平均水平的差距，制定“十二五”时期残疾人基本公共服务国家基本标准。

“十二五”时期残疾人基本公共服务国家基本标准

服务项目	服务对象	保障标准	支出责任	覆盖水平

残疾人社会保障

社会保险保费补贴	重度和贫困残疾人	参加城镇居民基本医疗保险、新型农村合作医疗、新型农村社会养老保险和城镇居民社会养老保险按规定享受政府社会保险费补贴	中央和地方财政共同负担	目标人群覆盖率100%

基本医疗保障医疗康复项目	参保残疾人	运动疗法、偏瘫肢体综合训练、脑瘫肢体综合训练、截瘫肢体综合训练、作业疗法、认知知觉功能康复训练、言语训练、吞咽功能障碍训练、日常生活能力评定等医疗康复项目纳入基本医疗保险范围	基本医疗保险基金支出	目标人群覆盖率100%

残疾人基本服务

义务教育阶段特殊教育	适龄残疾儿童、少年	在“两免一补”基础上，针对残疾学生特殊需要，进一步提高补助水平；大中城市不能到校上学的残疾儿童、少年接受送教上门服务	中央和地方财政共同负担	学龄残疾儿童少年接受义务教育比率达到90%

残疾人教育资助	家庭经济困难的残疾儿童、青少年	义务教育、学前教育和高中阶段教育寄宿生享受生活费用和特殊学习用品、教育训练补助；高中阶段教育学费、杂费、课本费免费	中央和地方财政共同负担	义务教育和高中阶段教育资助目标人群覆盖率100%，为5.14万人次贫困残疾儿童提供学前教育训练费和生活补助

残疾儿童抢救性康复	0-6岁残疾儿童	对接受手术、辅具配置和康复训练等服务提供资助	中央和地方财政共同负担	覆盖93万人（次）左右目标人群

残疾人就业服务	城乡有就业愿望的残疾人	免费在公共就业服务机构和基层劳动就业社会保障公共服务平台享有职业介绍、职业指导等就业服务；对就业困难残疾人提供就业援助；免费在残疾人就业服务机构享有就业信息发布、残疾人职业培训等服务	地方政府负责，中央财政适当补助	实现城镇残疾人新增就业100万，为100万农村贫困残疾人提供实用技术培训

残疾人文化服务	残疾人	能够收看到有字幕和手语的电视节目，在公共图书馆得到盲文和有声读物等阅读服务	中央和地方财政共同负担	各级公共图书馆设立盲人阅览室，配置盲文图书及有关阅读设备；省市两级电视台普遍开办手语节目；影视剧和电视节目加配字幕

残疾人体育健身服务	残疾人	免费享有体育健身指导服务	中央和地方财政共同负担	建立1200个残疾人体育健身示范点，经常参加体育健身的残疾人比率达到15%以上

　第三节　保障工程

针对残疾人基本公共服务的特殊性和专业性，实施残疾人基本公共服务保障工程，提升残疾人基本公共服务能力。

--残疾人康复和托养设施建设工程。建设一批残疾人康复设施，配备相应的设备和专业人员，全面开展康复医疗、功能训练、辅助器具适配、心理辅导、康复转介、残疾预防、知识普及和咨询等康复服务；支持一批示范性专业托养机构建设，实施“阳光家园”计划，增强托养服务能力。

--特殊教育学校建设工程。改扩建和新建一批特殊教育学校，添置必要的教学、生活和康复训练设施，使每个地级市和人口30万以上、残疾儿童少年较多的县（市、区）都至少有1所按国家标准建设的特殊教育学校。

　第十二章　促进城乡、区域基本公共服务均等化

按照推进基本公共服务均等化和实施主体功能区规划、国家区域发展战略的要求，逐步建立城乡一体化的基本公共服务制度，健全促进区域基本公共服务均等化的体制机制，促进公共服务资源在城乡、区域之间均衡配置，缩小基本公共服务水平差距。

　第一节　促进城乡基本公共服务均等化

--加强城乡基本公共服务规划一体化。涉及公共服务的各类规划，要贯彻区域覆盖、制度统筹的原则要求，以服务半径、服务人口为基本依据，打破城乡界限，统筹空间布局，制定实施城乡统一的基本公共服务设施配置和建设标准。

--推进城乡基本公共服务制度衔接。以制度统一为切入点，抓紧制定和实施统筹城乡基本公共服务制度的工作目标和阶段任务。鼓励各地开展统筹城乡基本公共服务制度改革试点，有条件的可率先把农村居民纳入城镇基本公共服务保障范围；暂不具备条件的，要注重缩小城乡服务水平差距，预留制度对接空间。

--加大农村基本公共服务支持力度。进一步加大公共资源向农村倾斜力度，新增预算内固定资产投资要优先投向农村基本公共服务项目。制定并推行各类机构服务项目及其规范标准，提高农村基层公共服务人员专业化水平。鼓励和引导城市优质公共服务资源向农村延伸，包括充分利用信息技术和流动服务等手段，促进农村共享城市优质公共服务资源。

--以输入地政府管理为主，加快建立农民工等流动人口基本公共服务制度，逐步实现基本公共服务由户籍人口向常住人口扩展。结合户籍管理制度改革和完善农村土地管理制度，逐步将基本公共服务领域各项法律法规和政策与户口性质相脱离，保障符合条件的外来人口与本地居民平等享有基本公共服务。积极探索多种有效方式，对符合条件的农民工及其子女，分阶段、有重点地纳入居住地基本公共服务保障范围。

　第二节　促进区域基本公共服务均等化

--推进落实主体功能区基本公共服务政策。对优化开发区域和重点开发区域，要根据工业化、城镇化需要，加强基本公共服务能力建设，使基本公共服务设施布局、供给规模与人口分布、环境交通相适应。对限制开发和禁止开发区域，要加大财政转移支付力度和财政投入，保障不因经济开发活动受限制而影响基本公共服务水平的提高。

--加大困难地区基本公共服务支持力度。加大对贫困地区、革命老区、民族地区、边疆地区和集中连片特殊困难地区的基本公共服务财政投入和公共资源配置力度，政府基本公共服务投资项目优先向这些地区倾斜。鼓励发达地区采用定向援助、对口支援和对口帮扶等多种形式，支持这些地区发展基本公共服务，并形成长效机制。

--建立健全区域基本公共服务均等化协调机制。加强国务院各部门与省级政府间的磋商协调，保持区域间基本公共服务范围和标准基本一致，推动相关制度和规则衔接，做好投资、财税、产业、土地和人口等政策的配套协调。健全地方政府为主、统一与分级相结合的公共服务管理体制，着力加强省级政府推进省域内基本公共服务均等化的统筹职能。适应区域一体化发展要求，完善现有各类区域协调机制，强化其促进区域内基本公共服务协作、资源共享、制度对接作用。鼓励和倡导长三角、珠三角等发达地区率先实现基本公共服务一体化。

　第十三章　增强公共财政保障能力

建立与经济发展和政府财力增长相适应的基本公共服务财政支出增长机制，切实增强各级财政特别是县级财政提供基本公共服务的保障能力。

　第一节　明确政府间事权和支出责任

--综合考虑法律规定、受益范围、成本效率、基层优先等因素，合理界定中央政府与地方政府的基本公共服务事权和支出责任，并逐步通过法律形式予以明确。中央政府主要负责制定国家基本公共服务标准和政策法规，提供涉及中央事权的基本公共服务，协调跨省（区、市）的基本公共服务问题，以及对各省级政府提供的基本公共服务进行监督、考核与问责。按照国家统一制度框架，省级政府主要负责制定本地区基本公共服务标准和地方政策法规，提供涉及地方事权的基本公共服务，以及对市级和县级政府提供的基本公共服务进行监督、考核与问责。市级和县级政府具体负责本地基本公共服务的提供以及对基本公共服务机构的监管。

--逐步将适合更高一级政府承担的事权和支出责任上移，增加中央和省级政府在基本公共服务领域的事权和支出责任。强化省级政府在教育、就业、社会保险、社会服务、医疗卫生等领域基本公共服务的支出责任。

　第二节　完善转移支付制度

--科学设置、合理搭配一般性转移支付和专项转移支付。在明确划分各级政府基本公共服务事权和支出责任的基础上，逐步做到属于地方政府事务，其自有收入不能满足支出需求的，中央财政原则上通过一般性转移支付给予补助；属于中央委托事务，中央财政通过专项转移支付足额安排资金；属于中央地方共同事务，明确各自支出的分担比例。

--完善转移支付办法。增加一般性转移支付特别是均衡性转移支付规模和比例，加大对中西部地区转移支付力度，优先弥补禁止开发区和限制开发区的收支缺口。规范专项转移支付，充分发挥专项转移支付资金促进基本公共服务均等化的积极作用。

--加快完善省以下转移支付制度。充分发挥省级财政转移支付有效调节省内基本公共服务财力差距的功能。已实施省直管县财政改革的地区，省级政府要根据本地区实际情况，加大对县级政府的转移支付力度。没有实施省直管县财政改革的地区，省、市级政府要采取多种方式，增加对县级政府的转移支付。

　第三节　健全财力保障机制

--完善公共财政预算，优化财政支出结构。各级政府要优先安排预算用于基本公共服务，并确保增长幅度与财力的增长相匹配、同基本公共服务需求相适应，推进实施按照地区常住人口安排基本公共服务支出。加快构建以政府为主导、充分体现社会公平的再分配调节机制。

--拓宽基本公共服务资金来源。继续安排中央资金，支持贫困地区和薄弱环节提高基本公共服务能力，地方各级政府特别是省级政府要安排相应资金。充分利用国际金融组织贷款等有效融资形式，拓宽政府筹资渠道，增加基本公共服务基础设施投入。加大国有资本经营预算用于基本公共服务的支出比重。扩大全国社会保障基金规模。

--提高县级财政保障基本公共服务能力。中央财政制定县级基本公共服务财力保障范围和保障标准，并根据相关政策和因素变化情况动态调整。省、市级财政要按照本行政区划内基本公共服务均等化的要求，逐步提高县级财政在省以下财力分配中的比重，帮助困难县（市、区）弥补基本财力缺口。县级政府要强化自我约束，科学统筹财力，规范预算管理。中央财政要完善县级财政保障基本公共服务的激励约束机制，根据基层工作实绩实施奖励。

　第十四章　创新供给模式

在坚持政府负责的前提下，充分发挥市场机制作用，推动基本公共服务提供主体和提供方式多元化，加快建立政府主导、社会参与、公办民办并举的基本公共服务供给模式。

　第一节　建立多元供给机制

--在政府实施有效监管、机构严格自律、社会加强监督的基础上，扩大基本公共服务面向社会资本开放的领域。各地区、各部门在制定规划和配置公共服务资源时，要给非公立机构留有合理空间，特别是配置新增资源时要统筹考虑由社会资本举办服务机构和提供服务。鼓励和引导社会资本参与基本公共服务设施建设和运营管理。公平开放基本公共服务准入，大力发展民办幼儿园和职业培训机构，鼓励和引导社会资本举办医疗机构和参与公立医院改制，推动社会资本兴办养（托）老服务和残疾人康复、托养服务等机构以及建设博物馆、体育场馆等文体设施。

--在实践证明有效的领域积极推行政府购买、特许经营、合同委托、服务外包、土地出让协议配建等提供基本公共服务的方式，抓紧研究制定分领域、分行业具体政策，包括规范准入标准、资质认定、登记审批、招投标、服务监管、奖励惩罚及退出等操作规则和管理办法。提供基本公共服务的民办机构，在设立条件、资质认定、职业资格与职称评定、税收政策和政府购买服务等方面，与事业单位享有平等待遇。

--充分发挥公共投入引导和调控作用，合理利用政府补贴供给方和补贴需求方的调节手段，探索财政资金对非公立基本公共服务机构的扶持，并积极采取财政直接补贴需求方的方式，增加公民享受服务的选择权和灵活性，促进基本公共服务机构公平竞争。

--提升社区基本公共服务能力，构建以社区为基础的城乡基层社会管理和公共服务平台。实施社区服务体系建设工程，以居民需求为导向，加强基层公共服务资源整合，因地制宜建设社区综合公共服务设施，行政办公、就业和社会保障、卫生计生、文化体育、科普宣传等设施加大共建共享力度。在外出就业较为集中的农村地区，要重点解决好留守家属的关爱服务，充分利用布局调整后闲置资源用于开展托老、托幼等服务。加快建设社会工作专业人才队伍，并建立专业人员引领志愿者服务的机制。

--提高基本公共服务信息化水平。积极构建国家数字化教学资源库和公共教育服务平台，加强就业、社会保险、基本社会服务、医疗卫生、人口和计划生育、保障性住房、文化体育等信息系统建设，促进信息资源整合共享。积极利用信息技术提高公共服务机构管理效率，创新服务模式和服务业态。

--逐步有序扩大基本公共服务领域对外开放，鼓励采用合资、合作等多种形式开展高水平的国际合作办医、养老以及文化体育等交流，鼓励中外合作办学。

　第二节　分类推进事业单位改革

--按照政事分开、事企分开和管办分离的要求，分类推进事业单位改革。对提供公共服务的事业单位，要强化公益属性，改革和完善政府投入方式，加强监督管理。承担义务教育、公共文化、公共卫生及基层的基本医疗服务等基本公益性服务，不能或不宜由市场配置资源的事业单位，划入公益一类；承担非营利医疗等公益服务，可部分由市场配置资源的事业单位，划入公益二类。

--探索管办分离的有效实现形式，完善法人治理结构，使事业单位真正转变为独立的事业单位法人和公共服务提供主体。积极推进体制改革，完善运行机制，配套推进机构编制、国有资产管理、人事管理、收入分配、社会保险改革。

　第三节　鼓励社会力量参与

--强化社会公众对基本公共服务供给决策及运营的知情权、参与权和监督权，健全基本公共服务需求表达机制和反馈机制，增加决策透明度。

--发挥各类社会组织在基本公共服务需求表达、服务供给与监督评价等方面的作用，把适合由社会承担的基本公共服务事项，以购买服务等方式交由社会组织承担。

--大力发展志愿服务，完善志愿服务管理制度和服务方式，促进志愿服务经常化、制度化和规范化，推动志愿服务与政府服务优势互补、有机融合。

--积极发展慈善事业，增强全社会慈善意识，积极培育慈善组织，完善慈善捐赠的法律法规和税收减免政策，充分发挥慈善在基本公共服务提供和筹资等方面的作用。

　第十五章　规划实施

本规划确定的目标和任务，是政府对人民群众的承诺，要切实加强组织领导和统筹协调，建立健全规划实施机制，全力确保完成。

　第一节　明确责任分工

本规划确定的各项指标和任务，要分解落实到国务院各有关部门和各省级人民政府。国务院各有关部门要按照职责分工，抓紧制定行业基本公共服务的具体标准，切实做好相关专项规划与本规划的衔接，并明确工作责任和进度。各省级人民政府要在国务院有关部门指导下，结合本地区实际，编制实施省级基本公共服务专项规划或行动计划，以国家基本标准为依据制定本地基本公共服务标准体系，并加强对市县级政府的绩效评价和监督问责。要建立高层次综合协调机制，协调解决规划实施中跨地区跨部门跨行业的重大问题。

各级政府要加大财力统筹，特别是中央财政和省级财政要合理确定与下级财政基本公共服务支出的分担比例，保证本规划确定的各项基本公共服务目标任务及保障工程的投入，保证本级财政承担的投入分年、足额落实到位。严格规范财政转移支付管理和使用，确保资金按时足额拨付。

　第二节　加强监督问责

发展改革委要加强对规划实施情况的跟踪分析，以开展全国基本公共服务水平综合评价为重要手段，制定评价指标体系和评价方案，牵头组织开展中期评估和终期评估，并向国务院提交评估报告，以适当方式向社会公布。

国务院各有关部门和各省级人民政府要开展本行业和本地区的基本公共服务水平监测评价，注意研究新情况，解决新问题。要自觉接受同级人大、政协和人民群众的监督。积极开展基本公共服务社会满意度调查。鼓励多方参与评估，积极引入第三方评估。

完善基本公共服务问责机制，增加基本公共服务绩效考核在政府和干部政绩考核中的权重。健全基本公共服务预算公开机制，增强预算透明度。切实加强对建设工程和专项拨款使用绩效的审计、监管。建立基本公共服务设施建设质量追溯制度，对学校、医院、福利机构、保障性住房等建筑质量实行终身负责制。
